# Ultra-Broadband Solar Absorption Enabled by 3D Crown-like Aluminum Nanostructure Arrays

**DOI:** 10.3390/nano16150904

**Published:** 2026-07-23

**Authors:** Yu Zhang, Xin Yan, Liqing Huang, Jun Wang, Lin Cheng, Yakun Cai, Huimin Wang, Weili Dong, Lipeng Zhai, You Liu, Jingping Zhu

**Affiliations:** 1 School of Physics, Xi’an Jiaotong University, Xi’an 710049, China; zhangyufengyun3@126.com (Y.Z.); 16635827379@163.com (X.Y.); smilekietty@126.com (L.C.); wuliyakun@163.com (Y.C.); whmlwhm@163.com (H.W.); 15902978596@163.com (W.D.); zhailp2018@xjtu.edu.cn (L.Z.); yoyossc@163.com (Y.L.); 2 School of Science, Xi’an Polytechnic University, Xi’an 710048, China; wjunxpu@126.com; 3 School of Electronic and Information Engineering, Xi’an Jiaotong University, Xi’an 710049, China; jpzhu@xjtu.edu.cn

**Keywords:** aluminum nanostructures, broadband solar absorber, surface plasmon resonance, magnetic resonance, graded-refractive-index

## Abstract

Plasmonic nanostructures offer a practical solution for solar-to-thermal conversion, yet simultaneously achieving ultra-broadband absorption, scalable fabrication, and long-term stability using earth-abundant aluminum remains difficult. In this work, we present a three-dimensional (3D) crown-like aluminum nanostructure absorber that achieves an experimental average absorption of 92% across the solar spectrum (200–2500 nm), with only 1.9% degradation in average absorption over 24 months. The structure is fabricated via a scalable anodic aluminum oxide (AAO) template-assisted method, enabling large-area production without costly lithography and exhibiting broad fabrication tolerance to deposition-thickness variations. Electromagnetic simulations and structure analysis reveal that the ultra-broadband absorption arises from three synergistic mechanisms: multi-mode electric resonances, magnetic resonance behavior within the metal–dielectric–metal architecture, and a graded-refractive-index profile. Proof-of-concept photothermal experiments under simulated sunlight offer experimental confirmation of the absorber’s solar-to-thermal conversion capability, showing substantially enhanced solar-to-thermal energy utilization compared to pure-water references. This work provides a scalable, durable, and cost-effective platform for ultra-broadband solar absorption and solar-to-thermal conversion, and offers a viable design strategy for plasmonic absorbers based on earth-abundant materials.

## 1. Introduction

Efficient harvesting and conversion of solar energy are central to addressing global energy demands and environmental challenges. Surface plasmon polaritons (SPPs) are coherent oscillations of free electrons at metal–dielectric interfaces, coupled with electromagnetic waves. They offer an effective way to concentrate light at the nanoscale [1,2,3,4]. When incident photons interact with metallic nanostructures at resonant frequencies, localized surface plasmon resonances (LSPRs) are excited, producing strong near-field enhancement and efficient light-matter interactions [5,6,7]. This plasmonic enhancement enables strong absorption of electromagnetic radiation in subwavelength volumes, making plasmonic nanostructures highly attractive for solar energy harvesting applications, such as solar thermal conversion [8,9], photocatalysis [10,11], and photodetection [12,13].

Plasmonic solar absorbers exploit surface plasmon resonances to convert sunlight into heat [14], yet a central challenge lies in achieving broadband absorption across the full solar spectrum (200–2500 nm). Early absorbers based on two-dimensional (2D) nanostructures, such as nanodisks, nanorings, and nanohole arrays, typically exhibit narrow absorption peaks tied to specific resonance modes [15,16,17,18], as their limited confinement geometry supports only a few resonances [19]. Multi-layer stacking and hybrid designs can broaden the bandwidth, but often at the cost of increased fabrication complexity and reduced scalability [20,21]. Three-dimensional (3D) nanostructures overcome these limitations by supporting multiple plasmonic resonance modes, while the vertical dimension enables extended optical path lengths, enhanced light trapping, and the excitation of both electric and magnetic resonances [22,23,24]. Various geometries, such as, nanopillars, nanohemispheres, nanocones, and nanopyramids, have been shown to create gradient effective refractive indices that suppress reflection through impedance matching [24,25,26,27,28,29,30]. It should be noted that a graded index alone does not guarantee broadband performance—poorly designed 3D architectures may introduce unwanted scattering or fail to achieve impedance matching. Effective design therefore requires deliberate engineering to ensure that multiple resonance modes operate synergistically. To mitigate these design challenges, many studies resort to noble metals such as gold and silver, whose strong plasmonic responses offer greater design flexibility. However, the high cost of gold and the susceptibility of silver to oxidation and sulfidation hinder their scalability for large-area applications [31,32,33,34,35].

Aluminum has emerged as an attractive alternative plasmonic material due to its natural abundance, low cost, and excellent chemical stability, with a self-passivating native oxide layer (Al_2_O_3_, 2–3 nm) that ensures long-term durability [36,37,38]. Its plasmonic resonances extend from the ultraviolet to the near-infrared, making it particularly suitable for broadband solar absorption [39,40]. Recent studies have demonstrated aluminum-based nanostructures with absorption performance comparable to or exceeding that of noble metal counterparts. For instance, Zhai et al. fabricated broadband absorber base on three-dimensional aluminum nanospike arrays for hot electron-based photodetection, yet the broadband absorption relies heavily on an additional Au film [41]. Cheng et al. reported large-scale 3D Al/AlN plasmonic nanostructures achieving 96.8% absorption via reactive magnetron sputtering [42]. However, the incorporation of nitrogen introduces an additional element and increases fabrication complexity. While these works demonstrate the potential of aluminum-based plasmonics, their reliance on auxiliary materials or multi-element fabrication diverges from the goal of a purely aluminum-based, low-cost solution. To overcome these limitations, AAO template-assisted fabrication has emerged as a promising route toward scalable and cost-effective production of aluminum nanostructures [43]. Ryu et al. demonstrated a metal-coated Al_2_O_3_ nanowire bundle absorber fabricated via the controlled pore widening of AAO with multiscale funnel structures [44]; Zhou et al. reported 3D self-assembly of aluminum nanoparticles supported by AAO for plasmon-enhanced solar desalination with >96% solar absorption [45]. However, in both cases, the AAO template serves merely as a supporting scaffold rather than an active optical component, and the absorption mechanism relies predominantly on a single plasmonic mode rather than the synergistic integration of multiple resonance effects. Consequently, these designs do not fully exploit the potential of AAO-based architectures for broadband absorption across the entire solar spectrum. Therefore, aluminum nanostructures capable of synergistically integrating multiple optical mechanisms within a scalable AAO-based architecture are highly desirable for practical solar-energy-harvesting applications.

In this work, we demonstrate a 3D crown-like aluminum nanostructure absorber designed to overcome the spectral and cost limitations of traditional plasmonic systems. By leveraging a scalable tip-like ultrathin anodic aluminum oxide (TUAAO) template-assisted method, we fabricated a multi-scale crown-like architecture that achieves a measured average absorption of 92% across the 200–2500 nm range, with only 1.9% degradation over 24 months of ambient exposure. Notably, the crown-like aluminum nanostructure absorber exhibits broad fabrication tolerance, maintaining an average absorption above 89% over a deposition-thickness window of 100–140 nm. This tolerance is particularly significant for practical applications, as the AAO template-assisted method has been established as a scalable and cost-effective nanofabrication route [43]. Our results further demonstrate that the crown-like architecture relaxes the tight process-control requirements typically associated with plasmonic nanostructures, enhancing its suitability for reproducible, large-area manufacturing. Unlike previous AAO-based absorbers that use the template merely as a sacrificial scaffold [41] or as a supporting scaffold for depositing continuous metal films [44,45], our approach retains the TUAAO template as an integral part of the structure and produces discrete Al nanoparticles on both nanotips and barrier layer, enabling the synergistic operation of multiple optical mechanisms within a single scalable architecture. To elucidate the physical origin of these mechanisms, finite-difference time-domain (FDTD) simulations were performed to resolve the electromagnetic responses within the crown-like architecture. The analysis reveals that the exceptional broadband absorption originates from the synergistic operation of three complementary mechanisms: multi-mode electric resonances (localized surface plasmons, gap surface plasmons, and cavity modes) spanning the ultraviolet to near-infrared regions; magnetic-resonance behavior sustained within the alumina barrier layer of the metal–dielectric–metal structure, which provides an additional absorption channel in the near-infrared regime; and a graded-refractive-index effect that suppresses broadband reflection through impedance matching. Prototype photothermal experiments under simulated (1 sun and 3 suns) sunlight conditions provide practical validation of the absorber’s solar-to-thermal conversion capability, demonstrating significantly enhanced thermal response and solar-to-thermal energy utilization compared with pure-water references. By integrating scalable fabrication, electromagnetic modeling, and photothermal validation, this study elucidates the synergistic mechanisms responsible for ultra-broadband absorption in crown-like aluminum nanostructures and demonstrates their potential for efficient solar-to-thermal conversion.

## 2. Materials and Methods

### 2.1. Fabrication of TUAAO Template and Crown-like Al Nanostructure Arrays

**Figure 1:**
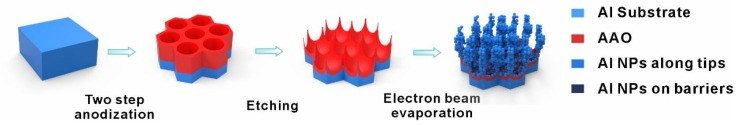
Schematic illustration of the fabrication process for the Al crown-like nanostructure. The procedure involves a two-step anodization to form the AAO substrate, followed by controlled phosphoric acid etching to create TUAAO template. The final 3D crown-like architecture is realized through electron beam evaporation of aluminum, resulting in a hierarchical distribution of Al nanoparticles along the tips and upon the barriers.

The 3D crown-like Al nanostructure was fabricated using an anodic aluminum oxide (AAO) template-based method, as illustrated in Figure 1. This approach can create highly ordered nanostructure arrays over large areas without the need for expensive lithography.

The process began with the preparation of the tip-like ultrathin anodic aluminum oxide (TUAAO) membrane, via two-step anodization [46] and wet chemical etching. In the first anodization step, a mirror-electropolished aluminum substrate (99.999% purity, 0.2 mm thickness, purchased from Trillion Metals, Beijing, China) was anodized in a 0.3 M oxalic acid (H_2_C_2_O_4_) (purchased from Shanghai Aladdin Biochemical Technology Co., Ltd., Shanghai China,) solution at a constant voltage of 120 V and a temperature of −1.5 °C for 1 h. The resulting oxide layer was subsequently removed by etching in a mixed solution of 5 wt% phosphoric acid (H_3_PO_4_) (purchased from Shanghai Aladdin Biochemical Technology Co., Ltd., Shanghai China) and 1.8 wt% chromic acid (H_2_CrO_4_) (purchased from Shanghai Aladdin Biochemical Technology Co., Ltd., Shanghai China) at 60 °C for 12 h to create highly ordered surface indentations. In the second anodization step, the pre-textured substrate was anodized in a 0.3 M phosphoric acid (H_3_PO_4_) solution at a constant voltage of 100 V and a temperature of 3 °C for 20 min. This specific voltage was selected to match the period of the ordered indentations (~250 nm) inherited from the first anodization step under oxalic acid [47], ensuring the ordered growth of the newly formed AAO in the second step. The TUAAO membrane was then formed by controlled etching in a 5 wt% phosphoric acid solution at 35 °C for 70 min, yielding a periodic array of alumina nanobowls with nanoscale tips at the intersection points of three neighboring nanobowls. The TUAAO template contained the TUAAO membrane and the remaining aluminum substrate.

Finally, the crown-like Al nanostructure arrays were fabricated by electron beam (E-beam) (EB700, SKY Technology Development Co.,Ltd. Shenyang, China) evaporation of aluminum onto the TUAAO template under a high vacuum of approximately 1 × 10^−3^ Pa. Through optimization of the deposition parameters, a deposition thickness of 120 nm was identified as the optimal condition, yielding the highest broadband absorption (92%; see Section 3.2). The evaporation rate (0.1 nm/s) and thickness were monitored in real time by the film thickness controller (quartz crystals inside). During the evaporation process, the template temperature was maintained at 350 °C to facilitate the surface diffusion and coalescence of aluminum grains [48], thereby ensuring the formation of the discrete nanoparticle distribution that is critical for the crown-like architecture. This elevated temperature is essential, as insufficient thermal energy would instead lead to the formation of a continuous film.

### 2.2. Characterization of TUAAO Template and Crown-like Al Nanostructure Arrays

The morphological evolution of the TUAAO template and the crown-like Al nanostructure arrays was characterized using a field-emission scanning electron microscope (FE-SEM, JSM-6700F, JEOL, Tokyo, Japan). Both top-view and tilted-view images were captured to accurately determine the structural dimensions, including the periodicity, nanotip height, and the spatial distribution of the deposited Al nanoparticles. Optical characterization was performed by measuring the total hemispherical reflectance spectra (R(λ)) in the UV–Vis–NIR range (200–2500 nm) using a high-performance UV–Vis–NIR spectrophotometer (Lambda 750, PerkinElmer, Waltham, MA, USA). The system was equipped with a 60 mm diameter barium sulfate (BaSO_4_) coated integrating sphere to capture both specular and diffuse reflections. A calibrated BaSO_4_ plate served as the 100% reflectance standard, and all measurements were conducted at a near-normal incident angle of 8°. Because the bottom aluminum substrate is sufficiently thick (~0.2 mm) to render the structure optically opaque (T(λ) = 0%), the absorption spectra (A(λ)) were derived according to the relation: A(λ) = 100% − R(λ). The spectral average absorbance was calculated by the integral mean of the absorption spectra (A(λ)) over the entire wavelength range, see Section S1, Supplementary Material. This approach ensures that the calculated absorption directly represents the intrinsic energy-harvesting capability of the crown-like architecture.

### 2.3. Electromagnetic Simulation of TUAAO Template and Crown-like Al Nanostructure Arrays

To systematically elucidate the physical origins of the ultra-broadband absorption, three-dimensional finite-difference time-domain (FDTD) simulations were performed using a commercial software package (Ansys Lumerical FDTD, version 8.19, Pittsburgh, PA, USA). The simulation domain was defined by a unit cell with a periodic boundary condition (PBC) in the x and y directions and a perfectly matched layer (PML) in the z direction to simulate an infinite array. The geometric model (see Figure S2, Supplementary Material, for the unit cell model) was constructed based on the SEM images. A non-uniform mesh grid was employed, with a refined mesh size of 1 nm within the nanostructure region. The plane wave source with a spectral range of 200–2500 nm normally incident along the *z*-axis was employed. Frequency-domain field profile monitors were placed to extract the normalized electric and magnetic field distributions (|E|/|E_0_| and |H|/|H_0_|). The induced current vectors were derived via post-processing of the simulated electromagnetic field data using Maxwell’s equations. The complex dielectric functions for aluminum and alumina were obtained from Palik’s experimental data [49].

### 2.4. Proof-of-Concept Experiments of Solar-to-Thermal Conversion

To evaluate the solar energy harvesting and transduction capabilities of the Al crown-like nanostructure, photothermal conversion experiments were conducted in laboratory conditions. A xenon lamp (CEL-HXF300, CEAuLight, Beijing, China) was utilized as the simulated solar source. The incident optical intensity was calibrated to ~1000 W/m^2^ (1 sun) and ~3000 W/m^2^ (3 suns) using a standard optical power meter (VLP-2000, Ranbond, Beijing, China). The experimental configuration consisted of a quartz container (inner diameter: 4 cm) containing 10 g of deionized water, with the Al crown-like absorber (diameter: 3.5 cm) positioned at the bottom, as shown in Figure S3 in Supplementary Material. A control group was established using an identical setup without the Al crown-like absorber. Real-time temperature of the water body was measured using a high-precision thermocouple and infrared thermal imager (FLUKE, Everett, WA, USA). The total photothermal utilization efficiency was derived via energy balance analysis. This calculation integrated the sensible heat storage, determined by the temperature lift, and the latent heat of evaporation, determined by mass change measurements using an electronic balance. The specific results see Section 3.3.

## 3. Results and Discussion

### 3.1. Morphology and Optical Characteristics of the TUAAO Template and Crown-like Al Nanostructure Arrays

**Figure 2:**
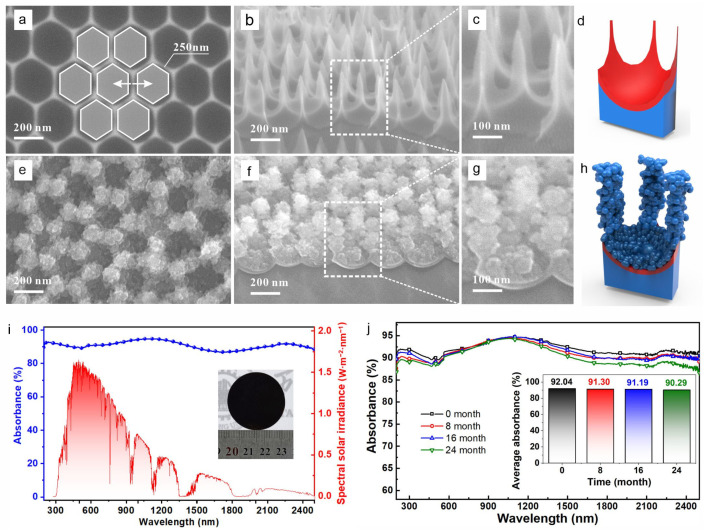
Morphological and optical characterization of the TUAAO template and the optimized crown-like Al nanostructure. (**a**–**c**) Top-view, tilted-view, and high-magnification SEM images of the bare TUAAO template showing the hexagonal close-packing. (**d**) Corresponding 3D geometric model. (**e**–**g**) Top-view, tilted-view, and high-magnification SEM images of the optimized crown-like Al nanostructure (120 nm Al-deposited) showing the well-defined 3D crown-like structure. (**h**) Corresponding 3D geometric model. (**i**) Experimental absorption spectrum of the optimized Al crown-like nanostructure, overlaid with the AM 1.5 G solar spectrum, and a photograph of the sample. (**j**) Long-term optical stability measurement over 24 months, showing negligible degradation in average absorption under ambient conditions.

The morphology of the TUAAO template and the optimized crown-like Al nanostructure (120 nm deposition of aluminum) is presented in Figure 2. As shown in the SEM image (Figure 2a–c), the two-step anodization and wet chemical etching process produces a highly ordered, hexagonally close-packed periodic array with nano-tips, namely the TUAAO template. The structural period is measured at 250 nm, which is consistent with the theoretical expectations for anodization in oxalic acid at 120 V. Upon deposition of 120 nm of aluminum, the template transforms into a well-defined 3D crown-like architecture. Tilted-view SEM imaging (Figure 2f,g) shows that Al nanoparticles are distributed along the Al_2_O_3_ nanotips and over the barrier layer. The original nanotip height of 390 nm, combined with the accumulation of Al grains, results in a total structural height of approximately 600 nm. This multi-scale geometry is critical for the intended optical performance: the vertical scaffolding provides the necessary degrees of freedom for 3D light trapping, while the discrete Al nanoparticle distribution creates a spatially varying filling fraction. This architecture effectively establishes the gradient refractive index required for broadband impedance matching, as further discussed in the following sections.

The optimized crown-like Al nanostructure (120 nm deposition of aluminum) achieves an experimental spectral average absorption of 92.0% over the 200–2500 nm wavelength range. As shown in Figure 2i, the absorption spectrum well matches the AM 1.5 G solar irradiance profile, demonstrating that the structure can efficiently capture solar energy from ultraviolet to near infrared. Furthermore, this high absorption level is sustained even after prolonged exposure to ambient air. Figure 2j reveals only 1.9% degradation in average absorption over a 24-month period, confirming the exceptional long-term optical stability. Optical absorption is highly sensitive to morphological changes. The stable optical performance over 24 months therefore strongly indicates that the nanostructure remains largely intact under ambient conditions. The self-passivating Al_2_O_3_ layer naturally formed on the aluminum surface plays a key role in preventing performance degradation and morphology transition caused by oxidative damage. Future investigations employing advanced topographical characterization techniques, such as AFM-based fractal analysis [50], could provide more quantitative insights into the correlation between structural preservation and optical performance.

To contextualize the performance of our absorber within the broader literature, we present in Table 1 a comparison with recently reported plasmonic absorbers.

### 3.2. Synergistic Evolution of Morphology and Optical Absorption with Deposition Thickness

**Figure 3:**
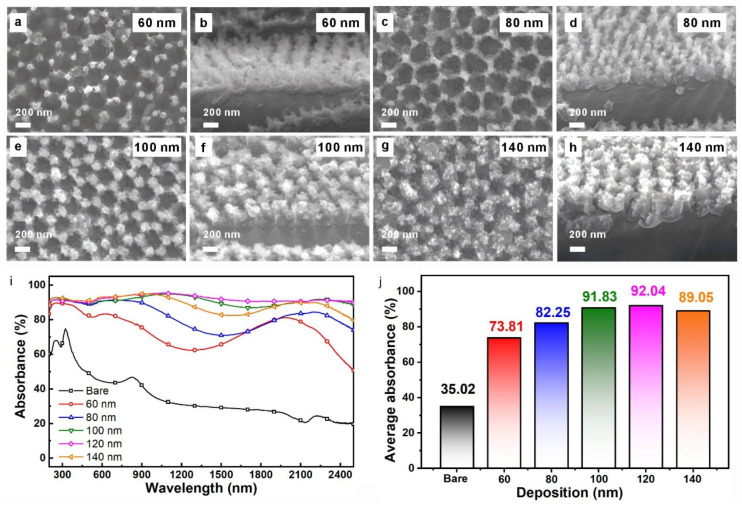
Morphological and optical evolution as a function of Al deposition thickness. (**a**–**h**) SEM images showing the structural transformation from sparse particles to coalesced clusters across thicknesses of 60 nm, 80 nm, 100 nm, and 140 nm. (**i**,**j**) Experimental absorption spectra and average absorption values of the TUAAO template and Al nanostructures with different deposition thicknesses, confirming 120 nm as the optimal deposition thickness.

The optical performance of the crown-like Al nanostructure is intrinsically linked to its morphological evolution, which is determined by the Al deposition thickness. Figure 3 shows how the optical absorption properties correlate with structure across various Al deposition thicknesses (60–140 nm).

At the lowest deposition thicknesses of 60–80 nm, the Al particles deposited on the Al_2_O_3_ nanotips were sparse and isolated (Figure 3a–d). This incomplete coverage fails to support a continuous plasmonic network, resulting in relatively low average absorptions of 73.8% and 82.3% (Figure 3j), respectively. As the thickness increases to 100 nm, the particle density and size grow (Figure 3e,f), facilitating stronger near-field coupling and the emergence of gap plasmon modes. This is reflected in the steady rise in average absorption to 90.8%, as multiple resonance modes begin to overlap across the Vis-NIR spectrum. The optimal performance is achieved at a thickness of 120 nm, where a well-defined crown-like architecture is formed with ideal particle spacing and distribution. The detailed morphology of this optimized 120 nm sample, serving as the performance benchmark in Figure 3i, is previously illustrated in Figure 2e–g. This configuration yields a remarkable average absorption of 92.0% over the 200–2500 nm range, with the absorption remaining consistently high (>89%) throughout the entire range, indicating the simultaneous excitation of electric, magnetic, and cavity modes. Further increasing the thickness to 140 nm leads to a moderate decrease in average absorption to 89.0%. Although the absolute absorption remains relatively high, the spectral flatness degrades noticeably. This degradation occurs because the coalescence of adjacent Al particles (Figure 3g,h) reduces the density of discrete plasmonic hotspots and weakens the near-field inter-particle coupling that is essential for sustaining multiple resonance modes across the broadband spectrum. Meanwhile, the top surface becomes more uniformly metallic instead of a sparse array of nanotips, leading to a higher effective refractive index at the top surface and thus stronger Fresnel reflection. The decline in performance at 140 nm confirms 120 nm as the optimal deposition thickness. This regular evolution of absorption with deposition thickness demonstrates that broadband optical performance is highly sensitive to the morphology of the structure. To quantitatively characterize the morphological evolution, we analyzed the pore size distribution of the AAO template at different Al deposition thicknesses from SEM images (see Figure S4, Supplementary Materials). The average pore diameter decreases from 205 nm to 146 nm as the deposition thickness increases from 60 nm to 140 nm, confirming the progressive filling and coalescence of nanoparticles within the template pores. This quantitative trend correlates well with the optical absorption evolution shown in Figure 3j. In particular, the superior performance of the 120 nm sample suggests that an optimized balance between particle coverage, inter-particle spacing, and hierarchical structural features is achieved at this thickness. These observations imply that multiple optical processes may operate simultaneously within the crown-like structure. To elucidate the physical origin of the ultra-broadband absorption, detailed electromagnetic simulations were subsequently performed and are discussed in the next section.

Notably, the average absorption remains above 89% across a broad deposition-thickness range of 100–140 nm, despite substantial morphological evolution of the Al nanoparticle network. Such behavior indicates that the optical performance is not critically dependent on a narrowly defined geometry, but instead benefits from the collective contribution of multiple absorption mechanisms. This robust fabrication tolerance relaxes process-control requirements, improves reproducibility, and enhances the feasibility of large-area manufacturing.

### 3.3. Physical Mechanism Analysis

To reveal the origin of the ultra-broadband absorption, three-dimensional FDTD simulations on the optimized 120 nm crown-like structure was performed. The agreement between the experimental and FDTD-simulated absorption spectra (Figure S5, Supplementary Material) validates the accuracy of the geometric model. Sensitivity analysis of geometric uncertainties confirms the robustness of the simulation model (see Figure S6, Supplementary Material). Building on this validated model, we analyzed the simulated electromagnetic field distributions and effective refractive index profiles to elucidate the physical mechanisms underlying the measured broadband absorption.

1. Multi-mode electric resonance: from isolated LSPR to cavity-enhanced absorption

**Figure 4:**
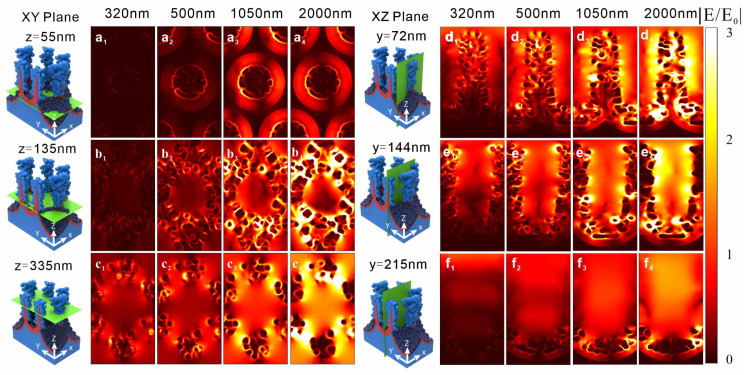
Simulated electric field intensity distribution characteristics. The XY Cross-sectional (**a_1_**–**c_4_**) and the XZ cross-sectional (**d_1_**–**f_4_**) (green plane in schematic) intensity profiles (|E|/|E_0_|) at representative wavelengths of 320 nm, 500 nm, 1050 nm, and 2000 nm, revealing the light-trapping mechanisms. The localized hot spots transition from individual nanoparticle tips (LSPR) at shorter wavelengths to gap-plasmon and cavity-like modes at longer wavelengths.

Various simulated electric field distribution (|E|/|E_0_|) profiles were extracted at different positions to reveal the electric field response characteristics across different regions of the 3D crown-like nanostructure. Figure 4a–c present cross-sectional views of the electric field distribution in the XY-plane at three representative locations along the vertical axis, while Figure 4d–f show the corresponding XZ-plane distributions across three horizontal positions. The characteristics of the field distribution reveal wavelength-dependent resonance mechanisms. At 320 and 500 nm, the field enhancement is predominantly localized at the surfaces of individual Al nanoparticles, with only minor enhancement in the inter-particle gaps, which is a characteristic of localized surface plasmon resonance (LSPR). As the wavelength increases to 1050 and 2000 nm, the field enhancement intensifies markedly in the gaps between adjacent Al nanoparticles and extends into the spaces between neighboring nanotips, indicating the excitation of gap surface plasmon polaritons (gap SPPs) driven by plasmon coupling across the nanogaps. In addition to these plasmon modes, the vertical gap between the Al nanotips and the Al backplane functions as a Fabry–Pérot-like cavity. At wavelengths satisfying the phase condition, constructive interference within this cavity further enhances the electric field, as evidenced by the interference features observed in Figure 4f. This cavity resonance operates in parallel with LSPR and gap SPPs, collectively contributing to the broadband electric-field-based absorption across the solar spectrum. This multi-mode operation, from isolated LSPR through gap SPPs to cavity resonance, ensures continuous electric-field-based absorption across the entire 200–2500 nm solar spectrum range.

2. Magnetic resonance behavior and current distribution

The 3D crown-like architecture inherently embeds a metal–dielectric–metal (MDM) configuration. The Al nanoparticle clusters distributed on the alumina barrier layer and the underlying Al substrate serve as the two metallic components, while the 30 nm Al_2_O_3_ barrier layer functions as the dielectric spacer. This MDM geometry provides a physical foundation for the excitation of magnetic resonance modes that are complementary to the electric resonances discussed above.

**Figure 5:**
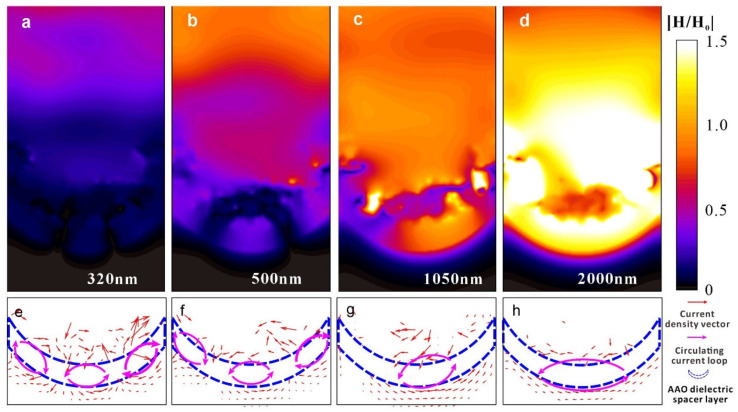
Magnetic-field confinement and current-loop analysis. (**a**–**d**) Magnetic field intensity profiles (|H|/|H_0_|) at 320 nm, 500 nm, 1050 nm, and 2000 nm, respectively. (**e**–**h**) Corresponding induced current vector plots near the AAO barrier layer. The circulating current loops and strong magnetic confinement within the Al_2_O_3_ barrier layer confirm the excitation of magnetic resonance in the NIR region.

To investigate the magnetic resonance behavior, we analyzed the simulated magnetic field intensity distributions (|H|/|H_0_|) and the corresponding induced current vectors. Figure 5a–d presents the simulated magnetic field intensity distributions (|H|/|H_0_|), while Figure 5e–h shows the corresponding induced current vectors around the AAO barrier at the same representative wavelengths. The cross-sectional plane is taken along the midlines between adjacent unit-cell vertices, capturing the flat barrier region where the large-area MDM response dominates. Complete magnetic field distributions across multiple cross-sectional planes are provided in Figure S7 (Supplementary Material). At 320 nm and 500 nm, the magnetic field enhancement is relatively weak, indicating that LSPR of the nanoparticles along the AAO nano-tips dominates the absorption in the UV–Vis region. At 1050 nm and 2000 nm, pronounced magnetic-field confinement emerges within the Al_2_O_3_ barrier layer. Simultaneously, the induced current vectors reveal well-defined circulating current loops, manifesting as anti-parallel conduction currents both in the Al nanoparticle clusters upon the Al_2_O_3_ barrier and in the underlying Al substrate. Such current-loop configurations are widely recognized as characteristic electromagnetic signatures of magnetic resonance in plasmonic MDM systems, resulting in strong confinement and enhancement of the magnetic field within the dielectric gap. The emergence of strong magnetic-field confinement in the AAO barrier at 1050 nm and 2000 nm suggests that magnetic resonance becomes increasingly important in where the electric plasmonic response of aluminum is comparatively weakened, helping to maintain the ultra-broadband absorption response across the solar spectrum. To quantitatively verify the contribution of the barrier layer to the NIR absorption, we performed an additional FDTD simulation on a modified structure in which the Al_2_O_3_ barrier layer was replaced with Al (see Figure S8, Supplementary Material). The significant decrease in NIR absorption upon elimination of the dielectric spacer confirms that the MDM configuration is indeed responsible for the magnetic-resonance-enhanced absorption in this spectral region.

3. Gradient refractive index and impedance matching

Beyond the electric and magnetic resonances described above, the crown-like architecture provides an additional broadband mechanism for suppressing reflection. The crown-like morphology creates a spatially graded filling fraction of aluminum along the vertical direction. Near the top of the structure, the Al-coated nanotips occupy only a small cross-sectional area within each horizontal plane, resulting in a low effective aluminum filling fraction and an effective refractive index close to that of air. Moving downward, the Al nanoparticle clusters deposited across the barrier layer form an increasingly continuous metallic coverage. The increasing volume fraction of Al, combined with the underlying Al_2_O_3_ barrier, gradually raises the effective refractive index. Below the barrier layer, the aluminum substrate provides a pure metallic material.

**Figure 6:**
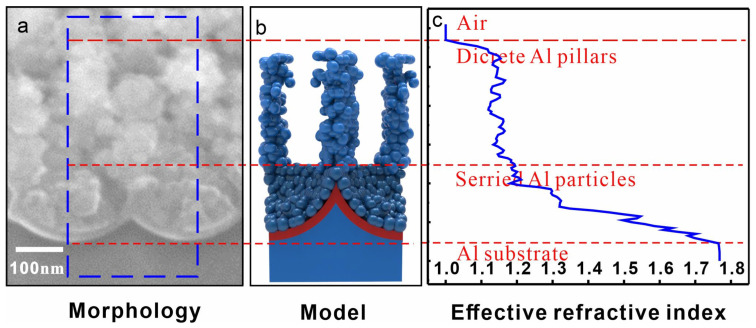
Impedance matching analysis via effective medium modeling. (**a**) Cross-sectional SEM image showing the vertical morphological gradient. (**b**) Schematic model used for effective refractive index calculations. (**c**) Calculated effective refractive index profile, showing a graded transition from the air to the metallic substrate that suppresses Fresnel reflection. The blue dashed box marks the exact region corresponding to the model, while the red dashed lines separate distinct refractive-index zones along the vertical direction.

To quantify the graded refractive index, we performed effective medium calculations based on the geometric model constructed from SEM images, as shown in Figure 6a,b. Horizontal cross-sections were taken at 5 nm intervals, and the effective refractive index of each slice was calculated using effective medium theory, in which the local permittivity at each pixel is determined by the material identity (air, Al_2_O_3_, or Al) and averaged over the plane. The detailed calculation method is described in Section S9 (Supplementary Material). The calculated refractive index profile, shown in Figure 6c, represents a simulation-based prediction that explains the broadband reflection suppression observed experimentally. The result exhibits a smooth transition from *n* ≈ 1.0 at the structure surface to *n* ≈ 1.8 at the alumina–aluminum substrate interface. This graded index bridges the optical impedance gap between air and the substrate, effectively eliminating an abrupt refractive index step. As a result, this continuous impedance matching suppresses Fresnel reflection across a broad spectral range. By providing a graded transition from the incident medium into the absorbing structure, this gradient-index design facilitates efficient photon injection across the entire 200–2500 nm range, ensuring that the incident solar radiation reaches the high-field regions where it is harvested by the electric and magnetic resonance mechanisms.

The combination of experimental absorption measurements and the simulation-based mechanistic analysis consistently supports the following conclusion. The measured 92% average absorption is therefore not attributable to any single mechanism in isolation. Rather, it emerges from the synergistic, spectrally complementary operation of all three. The graded-index anti-reflection layer operates as a broadband gateway, ensuring efficient photon injection across the full 200–2500 nm range. Once inside, isolated LSPRs on individual Al nanoparticles capture the UV–Vis photons, while gap SPPs driven by inter-particle plasmonic coupling extend the electric absorption into the near-infrared. In parallel, the magnetic resonance behavior within the MDM structure provides a physically distinct absorption channel in the NIR, ensuring robust performance where the electric response of aluminum diminishes. The crown-like morphology serves as the structural foundation of all three mechanisms, simultaneously providing the vertically graded filling fraction for impedance matching, the nanoscale gaps for gap SPP excitation, and the large-area MDM cavity for magnetic resonance. This mechanism-level complementarity is the physical basis of the ultra-broadband absorption across the solar spectrum. More importantly, it also helps explain the favorable tolerance to deposition-thickness variations observed in Figure 3i,j. Because the broadband absorption is not governed by a single narrowly defined resonance, moderate morphological changes mainly redistribute the relative contributions of LSPR, gap SPPs, cavity modes, magnetic resonance, and impedance matching, rather than eliminating the overall absorption pathways. As a result, high average absorption can be retained over a relatively broad deposition-thickness window, which is beneficial for reproducible and scalable fabrication.

### 3.4. Photothermal Validation of the Ultra-Broadband Absorber

To experimentally validate that the ultra-broadband optical absorption can be effectively converted into thermal energy, photothermal experiments were conducted under laboratory-simulated sunlight conditions. It should be emphasized that the present experiments are not intended to evaluate solar steam generation performance. Instead, the bottom-heating configuration was adopted to isolate the intrinsic solar-to-thermal conversion capability of the absorber and establish a direct relationship between broadband optical absorption and thermal energy generation. Unlike interfacial solar evaporators, the current system does not rely on capillary water transport or thermal localization at the air–water interface. Therefore, the photothermal experiments presented here should be regarded as a proof-of-concept validation of the absorber’s solar energy harvesting capability rather than an assessment of evaporation-device performance.

**Figure 7:**
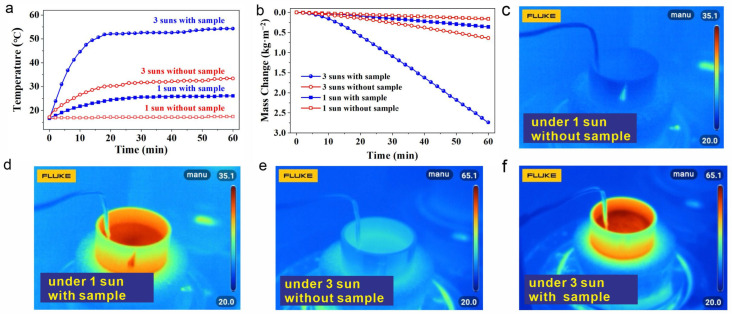
Photothermal conversion performance in the laboratory. (**a**) Temperature change curves of water with and without the Al absorber under 1 sun and 3 suns irradiation. (**b**) Corresponding mass change curves due to water evaporation. (**c**–**f**) Infrared thermal images of the samples and pure water under 1 sun (**c**,**d**) and 3 suns (**e**,**f**) after 60 min.

In the laboratory, photothermal conversion experiments were conducted under simulated solar intensities of 1 sun (1000 W/m^2^) and 3 suns (3000 W/m^2^) at an ambient temperature of ~17 °C. Due to the near-perfect solar absorption of 92.0% mentioned in Section 3.1, the absorber provides a robust physical foundation for efficient light-to-heat transduction. Under 1 sun irradiation, the water temperature integrated with the Al nanostructure rose from 16.9 °C to 26.1 °C within 60 min (ΔT = 9.2 °C), representing a significant enhancement over the pure water control group (from 16.6 °C to 17.4 °C, ΔT = 0.8 °C). Under 3 suns irradiation, the system achieved a final temperature of 52.6 °C (ΔT = 36.0 °C, from 16.6 °C), whereas the pure water only reached 33.4 °C (ΔT = 16.3 °C, from 17.1 °C), as shown in Figure 7a. The corresponding mass change curves (Figure 7b) confirm the enhanced evaporation performance of the Al absorber relative to the pure water reference. The infrared thermal images captured at the 60th minute (Figure 7c–f) display the temperature distribution across the entire water volume. Under both 1 sun and 3 suns illumination, the Al absorber groups exhibit a pronounced temperature elevation relative to the pure water references. The thermal energy is distributed relatively uniformly throughout the water body, which is a characteristic signature of the bottom-heating configuration.

To quantify the solar-to-thermal energy conversion capability of the absorber, a comprehensive energy-balance analysis was performed by considering both sensible heat storage in the water body and latent heat associated with evaporation. Under 1 sun irradiation, the Al crown-like absorber generated a total thermal output of 1266.9 J, consisting of 384.9 J of sensible heat and 882.0 J of latent heat from 0.36 g water loss, corresponding to a solar-to-thermal utilization efficiency of 36.6%. In comparison, the pure-water reference yielded only 425.5 J and an efficiency of 12.3%, indicating a 3.0-fold enhancement in thermal energy utilization. Under 3 suns irradiation, the total thermal output further increased to 8082.2 J, including 1506.2 J of sensible heat and 6576.0 J of latent heat associated with 2.74 g water loss, resulting in a solar-to-thermal utilization efficiency of 77.8%. By contrast, the pure-water reference generated 2218.0 J with an efficiency of 21.3%, corresponding to a 3.7-fold enhancement. The detailed calculation procedure and parameter values see Section S10 (Supplementary Material). The substantially high thermal output under different illumination demonstrates that the ultra-broadband absorber can efficiently harvest and convert incident solar radiation into useful thermal energy over a wide range of operating conditions. It should be noted that these efficiencies represent the overall solar-to-thermal energy utilization within the present bottom-heating configuration and should not be interpreted as solar steam generation efficiencies commonly reported for interfacial evaporation systems. Instead, they provide a quantitative assessment of the absorber’s intrinsic ability to convert broadband solar radiation into thermal energy.

## 4. Conclusions

In summary, a 3D crown-like aluminum nanostructure absorber was successfully fabricated using a scalable AAO template-assisted method. The optimized structure achieved an experimental average absorption of 92.0% over the 200–2500 nm solar spectrum and exhibited exceptional long-term optical stability, with only 1.9% degradation in average absorption after 24 months under ambient conditions, owing to the self-passivating Al_2_O_3_ layer. FDTD simulations revealed that the ultra-broadband absorption arises from the synergistic effects of three spectrally complementary mechanisms: multi-mode electric resonances, including isolated LSPRs, gap SPPs, and Fabry–Pérot-like cavity modes, that sequentially cover the ultraviolet to near-infrared range; a magnetic resonance behavior sustained within the Al_2_O_3_ barrier layer of the metal–dielectric–metal structure, which maintains absorption where the electric plasmonic response decays, and a graded-refractive-index effect that suppresses broadband reflection through impedance matching. These mechanisms enable efficient light harvesting across the entire solar spectrum. Experimental validation under simulated sunlight confirmed the effective conversion of the ultra-broadband optical absorption into thermal energy generation. The absorber demonstrated efficient conversion of broadband solar energy into thermal energy, achieving 3.0-fold and 3.7-fold higher solar-to-thermal energy utilization than the pure-water reference under 1 sun and 3 suns irradiation, respectively. The crown-like aluminum nanostructure combines high absorption efficiency, broad fabrication tolerance, strong durability, and scalable fabrication, providing a viable low-cost route for broadband solar energy harvesting based on earth-abundant materials.

## Figures and Tables

**Table 1:** Comparison of the crown-like Al nanostructure with previous reports.

Absorber Structure	Materials	Fabrication Method	Spectral Range	Absorption	Ref.
3D Crown-like Al nanostructure	Al	AAO template + E-beam evaporation	200–2500 nm	92.0% (avg)	This work
3D nanospike arrays	Al + Au	Anodization + Au deposition	400–800 nm	~90% (max)	Ref. [41]
3D Al/AlN nanostructures	Al + AlN	Reactive magnetron sputtering	300–2500 nm	94.2% (avg)	Ref. [42]
Self-aggregated Al funnel structures	Al	AAO template + sputter metal coating	300–2500 nm	84.5% (avg)	Ref. [44]
3D self-assembled Al NPs	Al	AAO template + physical vapour deposition	300–2500 nm	>96% (max)	Ref. [45]
Au nanotube with randomly sized	Au	AAO template + physical vapour deposition	300–800 nm	92% (max)	Ref. [34]
Au nanoporous films	Au	Chemical dealloying	300–2500 nm	91.4% (max)	Ref. [35]

## Data Availability

The original contributions presented in this study are included in the article/Supplementary Materials. Further inquiries can be directed to the corresponding authors.

## Supplementary Materials

The following supporting information can be downloaded at: https://www.mdpi.com/article/10.3390/nano16150904/s1. Section S1. Spectral average absorbance calculation method. Figure S2. FDTD unit cell model schematic. Figure S3. Photograph of the laboratory solar-to-thermal validation setup. Figure S4. Morphological and pore size evolution with Al deposition thickness. Figure S5. Comparison of experimental and simulated absorption spectra. Figure S6. Sensitivity analysis for geometric uncertainties of the simulated model. Figure S7. Complete magnetic field distributions (x-y and x-z cross-sections). Figure S8. Simulation for Decomposition analysis of magnetic resonance contribution. Section S9. Effective refractive index calculation method. Section S10. Total solar-to-thermal utilization efficiency calculation details.
