# Supplementary material for: Ultra-Broadband Solar Absorption Enabled by 3D Crown-like Aluminum Nanostructure Arrays
Source: Nanomaterials (Basel). 2026 Jul 23;16(15):904. doi: 10.3390/nano16150904 (PMC13467705; doi:10.3390/nano16150904)
Supplement: Supplementary file 1 [file nanomaterials-16-00904-s001.zip › nanomaterials-4411933-supplementary.pdf]

Supplementary material

Ultra-Broadband Solar Absorption Enabled by 3D Crown-Like Aluminum Nanostructure Arrays

Yu Zhang [1], Xin Yan [1], Liqing Huang [1,*], Jun Wang [2], Lin Cheng [1,2], Yakun Cai [1], Huimin Wang [1], Weili Dong [1], Lipeng Zhai [1], You Liu [1] and Jingping Zhu [3]

[1] School of Physics, Xi’an Jiaotong University, Xi'an 710049, China
[2] School of Science, Xi’an Polytechnic University, Xi'an 710048, China
[3] School of Electronic and Information Engineering, Xi’an Jiaotong University, Xi'an 710049, China
* Correspondence: lqhuangxjtu@126.com

- **Section S1.** Spectral average absorbance calculation method.
- **Figure S2.** FDTD unit cell model schematic.
- **Figure S3.** Photograph of the laboratory solar-to-thermal validation setup.
- **Figure S4.** Morphological and pore size evolution with Al deposition thickness.
- **Figure S5.** Comparison of experimental and simulated absorption spectra.
- **Figure S6.** Sensitivity analysis for geometric uncertainties of the simulated model.
- **Figure S7.** Complete magnetic field distributions (x-y and x-z cross-sections).
- **Figure S8**. Simulation for Decomposition analysis of magnetic resonance contribution.
- **Section S9.** Effective refractive index calculation method.
- **Section S10.** Total solar-to-thermal utilization efficiency calculation details.

S1. Spectral average absorbance calculation method

The spectral average absorbance is defined as the integral mean of the absorbance curve over the wavelength range, and its mathematical definition is:

$$\overline{A} = \frac{1}{\lambda_2 - \lambda_1} \int_{\lambda_1}^{\lambda_2} A(\lambda) d\lambda$$

In this work, the wavelength intervals of the data points in the measurement results are equal, so the calculation process can be simplified as:

$$\overline{A} = \frac{1}{N} \sum_{i=1}^{N} A_i$$

Where N is the total number of measured wavelengths and A_i is the absorbance of the corresponding wavelength.

S2. FDTD Simulation Model

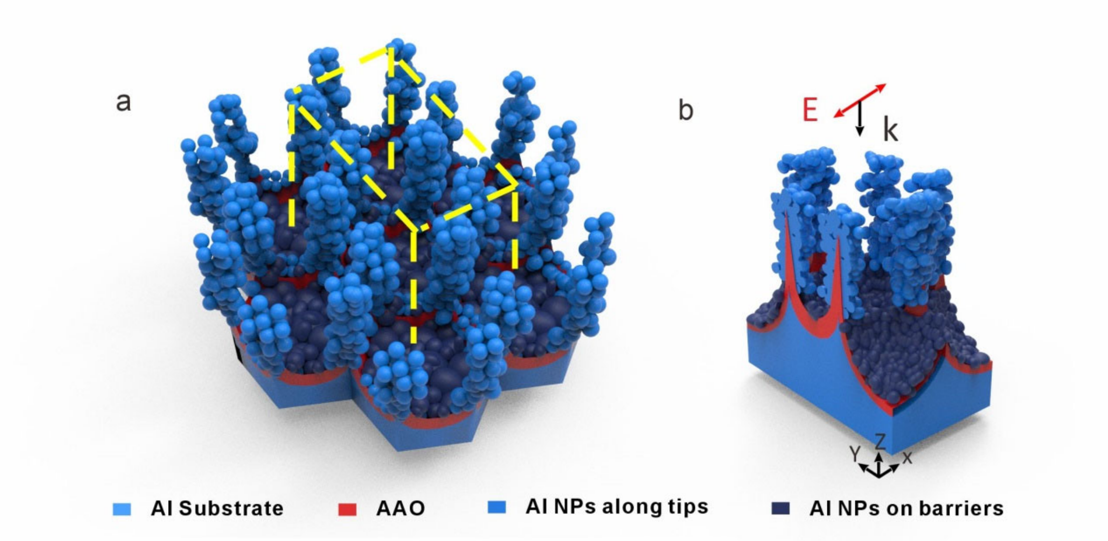

Figure S2. (a) Schematic of the Al crown-like nanostructure array, with the dashed box indicating one periodic unit cell used in the FDTD simulations. (b) Enlarged view of the unit cell model, showing the Al-coated nanotips, the Al particles on the Al_2O_2 barrier layer, the barrier layer itself, and the Al substrate. The structural period is 250 nm, the barrier layer thickness is 30 nm, and the nanotip height from the barrier layer top surface to the tip apex is approximately 390 nm. A conformal 3 nm native Al_2O_3 layer is included on all Al surfaces. The simulation domain employs periodic boundary conditions in the x and y directions and perfectly matched layers in the z direction.

S3. Photograph of the Laboratory Solar-to-Thermal Validation Setup

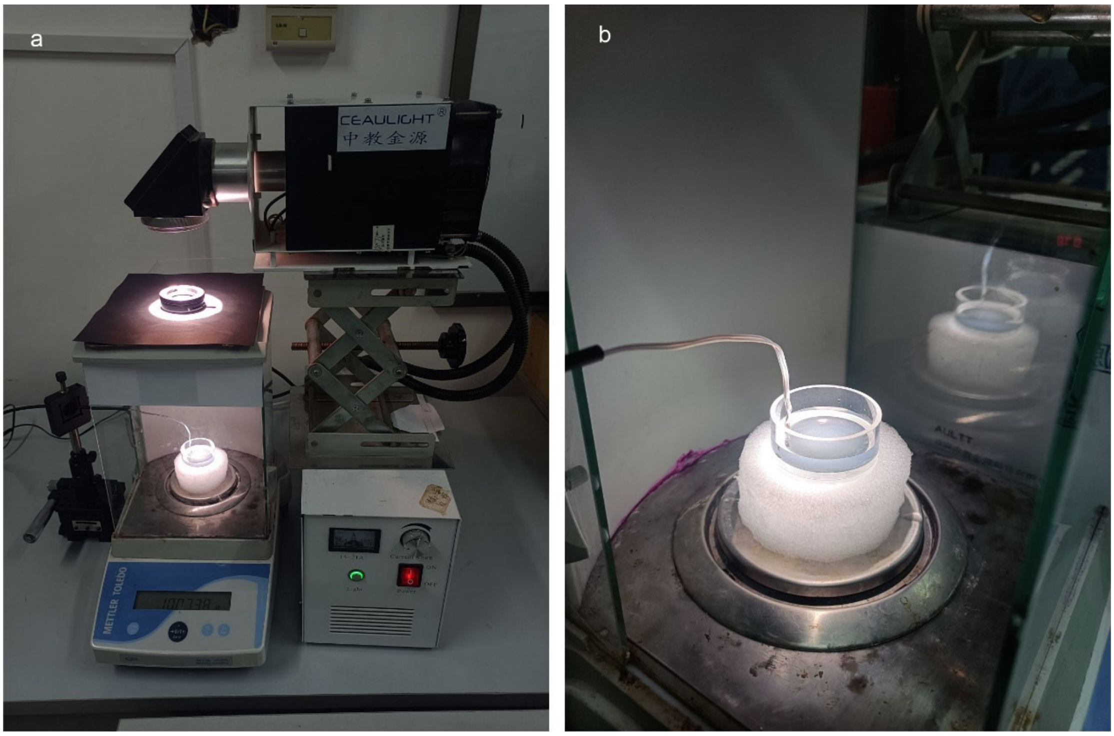

Figure S3. Photograph of the laboratory solar-to-thermal conversion experimental setup. The configuration consists of a quartz container (inner diameter: 4 cm) containing 10 g of deionized water, with the Al crown-like absorber (diameter: 3.5 cm) positioned at the bottom. A xenon lamp (CEL-HXF300) serves as the simulated solar source, calibrated to ~1000 W/m² (1 sun) or ~3000 W/m² (3 suns). Temperature evolution is monitored using a thermocouple and an infrared thermal camera (FLUKE). Real-time mass change was recorded using an electronic balance and used as part of the energy-balance analysis.

S4. Morphological and pore size evolution with Al deposition thickness

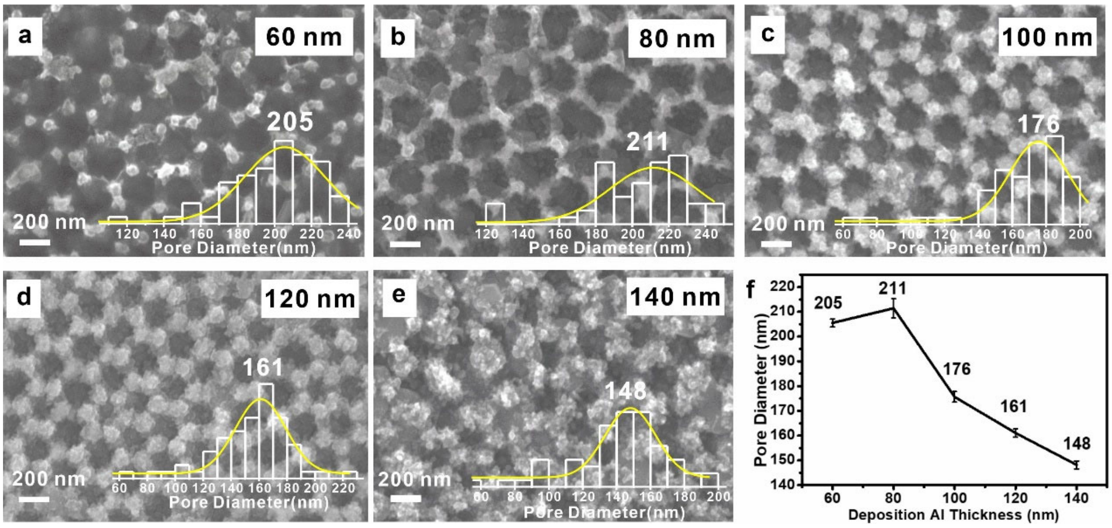

Figure S4. Morphological evolution with Al deposition thickness. (a–e) Top-view SEM images of AAO templates after Al deposition at thicknesses of 60, 80, 100, 120, and 140 nm, with pore size distribution histograms embedded. (f) Average pore diameter as a function of Al deposition thickness. The average pore diameters are 205, 211, 176, 161, and 146 nm for 60, 80, 100, 120, and 140 nm, respectively, showing a decrease with increasing thickness.

S5. Comparison of Experimental and Simulated Absorption Spectra

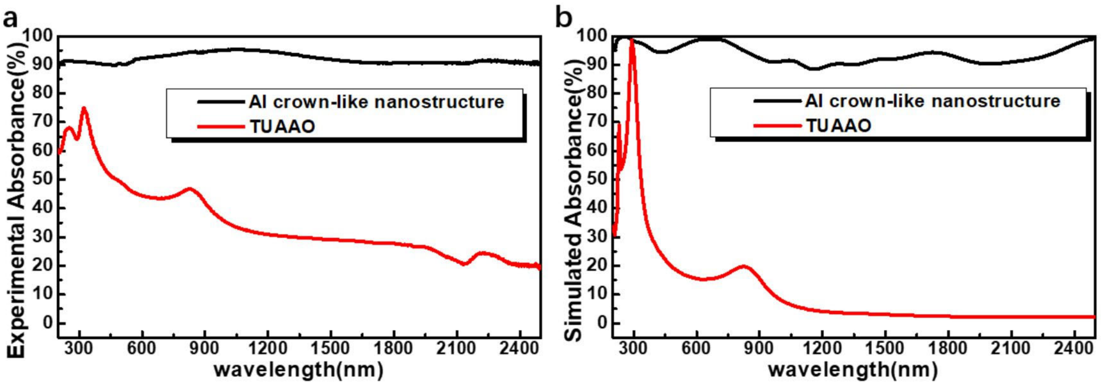

Figure S5. (a) Experimental absorption spectra of the optimized Al crown-like nanostructure (120 nm Al deposition; average absorption: 92.0%) and the bare TUAAO template (average absorption: 35.0%) measured over 200–2500 nm. The Al crown-like structure achieves near-perfect broadband absorption, whereas the bare TUAAO template shows significantly lower absorption due to the absence of plasmonic Al nanoparticles. (b) Corresponding FDTD-simulated absorption spectra for the same two structures (Al crown-like: 93.01%; TUAAO: 11.2%). The close agreement between the experimental and simulated spectra for the Al crown-like structure validates the accuracy of the geometric model constructed from SEM images. The discrepancy between the experimental (35.0%) and simulated (11.2%) TUAAO absorption is mainly attributed to residual metallic Al at the nanobowl bottom after the anodization/etching process, together with minor surface roughness not captured in the idealized simulation model. These factors introduce additional optical loss in the experimental TUAAO template, leading to higher measured absorption than that predicted by the simplified simulation.

S6. Sensitivity analysis for geometric uncertainties of the simulated model.

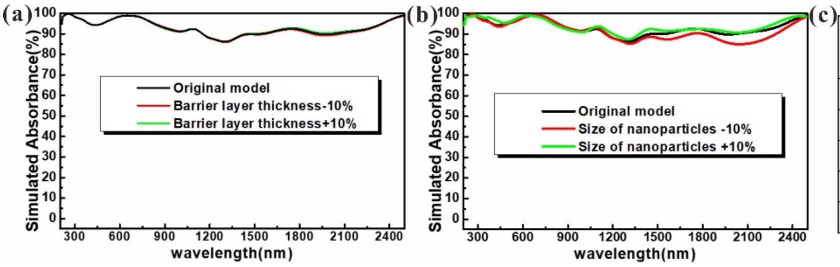

(c)

Change Geometric Parameters	Average Absorbtance (%)
original model	93.01
Barrier layer thickness -10%	92.86
Barrier layer thickness +10%	93.13
Size of nanoparticles -10%	91.5
Size of nanoparticles +10%	93.73

Figure S6. Sensitivity analysis of the FDTD simulation model. (a) Simulated absorption spectra with barrier layer thickness varied by ±10%. (b) Simulated absorption spectra with nanoparticle size varied by ±10%. (c) Summary of average absorption values for all five models: original (93.01%), barrier layer −10% (92.86%), barrier layer +10% (93.13%), nanoparticle size −10% (91.50%), nanoparticle size +10% (93.73%). The average absorption remains above 91% across all variations, confirming that the simulation conclusions are robust to reasonable geometric uncertainties.

S7. Complete Magnetic Field Distributions

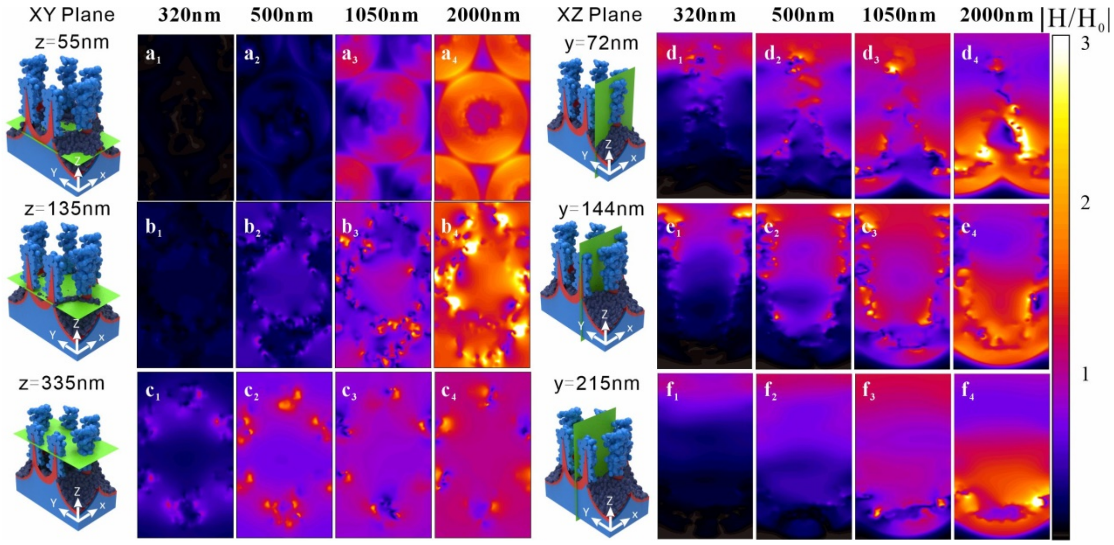

Figure S7. Complete simulated magnetic field intensity distributions ($|H|/|H_0|$) for the Al crown-like nanostructure at four representative wavelengths (320, 500, 1050, and 2000 nm). Left panel: x-y cross-sections at three different heights through the structure, with the section positions indicated on the unit cell model. Right panel: x-z cross-sections at three different y-positions, with the section positions similarly indicated. At 320 and 500 nm, magnetic field enhancement is relatively weak and distributed primarily around individual Al nanoparticles. At 1050 and 2000 nm, strong magnetic field confinement emerges within the Al_2O_3 barrier layer, consistent with the magnetic-resonance behavior within the MIM cavity discussed in Section 3.3. The x-z images at longer wavelengths clearly show the magnetic field concentrated within the flat barrier region between the Al particles on the barrier layer and the Al substrate, providing additional evidence for the large-area MIM response described in the main text.

S8. Simulation for Decomposition analysis of magnetic resonance contribution

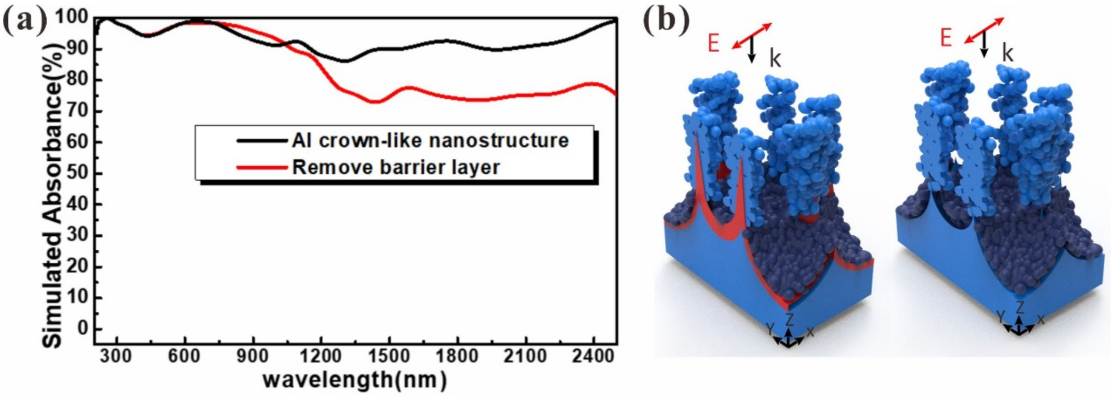

Figure S8. Decomposition analysis of magnetic resonance contribution. (a) Simulated absorption spectra of the original crown-like structure and the modified structure in which the Al_2O_3 barrier layer was replaced with Al. The absorption decreases significantly in the NIR region (1000–2500 nm) when the dielectric spacer is eliminated. (b) Schematic illustrations of the original model (left, with Al_2O_3 barrier layer) and the modified model (right, with Al barrier layer).

S9. Effective Refractive Index Calculation Method

The gradient-refractive-index profile presented in Figure 6c was calculated based on the FDTD geometric model constructed from SEM images. Horizontal cross-sections were taken at 5 nm intervals along the vertical (z) direction through the entire structure. Each cross-section was discretized into 1 nm × 1 nm pixels, and the material identity of each pixel—air (n = 1.0), Al_2O_3 (n ≈ 1.76), or Al (with the complex refractive index taken from the wavelength-dependent Palik data; n = 1.77 + 7.98i at 680 nm was used as a representative value for plotting the effective-index profile)—was assigned according to the spatial material distribution in the model. The effective refractive index profile at each height z was estimated by area-weighted averaging of the local refractive indices over all pixels within the corresponding cross-section:

$$n_{eff}(z) = \frac{1}{N}\sum_{i=1}^{N} n_i$$

Where N is the total number of pixels in the cross-section and n_i is the refractive index assigned to the i-th pixel. This procedure yields the continuous $n_{eff}(z)$ profile from the structure surface to the substrate, capturing the graded transition arising from the tapered Al filling fraction inherent to the crown-like morphology.

S10. Total Solar-to-Thermal Utilization Efficiency Calculation Details

The total solar-to-thermal utilization efficiency reported in Section 3.4 is defined as:

$$\eta = \frac{Q_{sensible} + Q_{latent}}{E_{in}}$$

where

- $Q_{sensible} = m \cdot c_p \cdot \Delta T$ is the sensible heat stored in the water body
- $Q_{latent} = \Delta m \cdot H_{vap}$ is the latent heat associated with water evaporation
- $E_{in} = P \cdot A \cdot t$ is the total incident solar energy

Parameter values:

Parameter	Symbol	Value
Water mass	m	0.010 kg
Specific heat capacity of water	c_p	4184 J/(kg·°C)
Latent heat of vaporization (25°C)	H_{vap}	2.45×10^6 J/kg
Latent heat of vaporization (50°C)	H_{vap}	2.40×10^6 J/kg
Illumination area (diameter 3.5 cm)	A	9.62×10^{-4} m^2
Illumination time	t	3600 s

1 sun results (P = 1000 W/m²):

Parameter	Al absorber	Pure water
Initial temperature	16.9 °C	16.6 °C
Final temperature	26.1 °C	17.4 °C
Temperature change, $\Delta \boldsymbol{T}$	9.2 °C	0.8 °C
Mass loss, $\Delta \boldsymbol{m}$	0.36 g	0.16 g
Sensible heat, $\boldsymbol{Q_{sensible}}$	384.9 J	33.5 J
Sensible heat, $\boldsymbol{Q_{latent}}$	882.0 J	392.0 J
Total thermal output, $\boldsymbol{Q_{total}}$	1266.9 J	425.5 J
Incident solar energy, $\boldsymbol{E_{in}}$	3463.2 J	3463.2 J
Solar-to-thermal utilization efficiency, $\boldsymbol{\eta}$	36.6%	12.3%

3 suns results (P = 3000 W/m²):

Parameter	Al absorber	Pure water
Initial temperature	16.6 °C	17.1 °C
Final temperature	52.6 °C	33.4 °C
Temperature change, $\Delta\boldsymbol{T}$	36.0 °C	16.3 °C
Mass loss, $\Delta\boldsymbol{m}$	2.74 g	0.64 g
Sensible heat, $\boldsymbol{Q_{sensible}}$	1506.2 J	682.0 J
Sensible heat, $\boldsymbol{Q_{latent}}$	6576.0 J	1536.0 J
Total thermal output, $\boldsymbol{Q_{total}}$	8082.2 J	2218.0 J
Incident solar energy, $\boldsymbol{E_{in}}$	10389.6 J	10389.6 J
Solar-to-thermal utilization efficiency, $\boldsymbol{\eta}$	77.8%	21.3%

Note: The latent heat of vaporization values were adjusted according to the different operating temperatures reached under 1 sun (~25 °C) and 3 suns (~50 °C). The calculated efficiencies represent the overall solar-to-thermal energy utilization in the present bottom-heating configuration.
